# Culture, executive functions, and academic achievement

**DOI:** 10.3389/fpsyg.2023.1100537

**Published:** 2023-05-12

**Authors:** Isu Cho, Niki Hosseini-Kamkar, Hyun-joo Song, J. Bruce Morton

**Affiliations:** ^1^Department of Psychology, Brandeis University, Waltham, MA, United States; ^2^Department of Psychiatry, McGill University, Montreal, QC, Canada; ^3^Department of Psychology, Yonsei University, Seoul, Republic of Korea; ^4^Department of Psychology, Western University, London, ON, Canada

**Keywords:** culture, executive functioning, academic achievement, control skills, model, social influences

## Abstract

Although it is well known that children of East Asian immigrants show higher academic achievement than native-born North American children, the social-cognitive determinants of this difference remain poorly understood. Given the importance of executive functions (EF) for academic achievement, and evidence that EF develops more quickly in East Asian compared to North American cultures, it is conceivable that differences in academic achievement might be rooted in EF differences between these groups. We examine this possibility by reviewing evidence of cross-cultural differences in EF development but find core concepts and findings limited in several key respects. To address these limitations, we propose a framework for relating EF, culture, and academic achievement that draws on new theoretical ideas about the nature of EF and its relation to social context. We conclude by discussing avenues for future research on the relations between culture, executive functions, and academic achievement.

## 1. Introduction

Children of East Asian immigrants outperform their native-born North American peers (Kao and Tienda, 2012; Feliciano and Lanuza, 2017; Tran C.D. et al., 2019; Kim, 2021; Xie, 2022) on several measures of academic achievement including the number of years of schooling completed (Keller and Tillman, 2008; Feliciano and Lanuza, 2017), performance on standardized proficiency tests (Glick and White, 2003; Duong et al., 2016), and grades (Hao and Woo, 2012; Kao and Tienda, 2012; Duong et al., 2016). Socio-cognitive factors that contribute to the East Asian advantage in academic achievement, however, remain poorly understood.

One possibility is that the advantage reflects differences in executive functioning (EF), domain-general cognitive processes that are critical for everyday psychological functioning. Across development, individual differences in EF predict academic achievement in math and reading both cross-sectionally (Altemeier et al., 2006; Blair and Razza, 2007; Brock et al., 2009; Mcauley et al., 2010; Willoughby et al., 2012; Thorell et al., 2013; Stipek and Valentino, 2015; Cortés Pascual et al., 2019; Nouwens et al., 2021) and longitudinally (Best et al., 2011; Jacob and Parkinson, 2015; Samuels et al., 2016; Ahmed et al., 2019; Robson et al., 2020; for review, see Zelazo and Carlson, 2020). Moreover, children from East Asian countries outperform North American and European children on a variety of EF measures, including measures of inhibition (Sabbagh et al., 2006; Oh and Lewis, 2008; Lan et al., 2011; Ellefson et al., 2017; Cho et al., 2021), cognitive flexibility (Sabbagh et al., 2006; Imada et al., 2013; Ellefson et al., 2017), and working memory (Oh and Lewis, 2008; Wang et al., 2016; Ellefson et al., 2017; but see Schirmbeck et al., 2021). This is true both of children born to East Asian immigrants (Cho et al., 2021) as well as children growing up in East Asian countries (Sabbagh et al., 2006; Oh and Lewis, 2008; Cho et al., 2021). Therefore, while it has not been tested directly, it is at least conceivable that the East Asian advantage in academic achievement stems, at least in part, from early differences in EF.

However, linking sociocultural background, EF, and academic achievement in this way arguably raises more questions than it answers. Why might East Asian children show early advantages in EF? Children from East Asian countries are subject to social and cultural influences that may be different from those experienced by Caucasian children in North America and Europe. For example, East Asian cultures are collectivist in nature because of their pronounced valuation of social harmony and the alignment of personal and communal goals. This contrasts with individualist Caucasian cultures of North America and Europe, which emphasize personal independence and self-expression (Tobin et al., 1989; Nisbett and Miyamoto, 2005; Tran V.C. et al., 2019). Parenting styles also differ across East Asian and Caucasian cultures of North America and Europe. East Asian parents tend to be authoritarian and “effort-oriented,” whereas Caucasian North American and European parents tend to be authoritative and encouraging of independence and personal expression (Stankov, 2010; Hsin and Xie, 2014; Kim, 2021; Boman, 2022). But how might differences in cultural values and socialization practices contribute to differences in children’s EF? Indeed, there is little consensus on whether children’s EF can be shaped by social experiences (for discussion, see Doebel, 2020). Even if we allow for social or cultural influences on children’s EF development, associations between social influences (such as parenting characteristics), and child outcomes can differ markedly across East Asian and Caucasian cultures of North America and Europe. The goal of the current paper is therefore twofold. First, to elaborate on some of the conceptual challenges related to understanding cross-cultural differences in EF development; and second, to propose a theoretical model linking culture, EF, and academic achievement.

## 2. Conceptual challenges to linking executive functioning development and social experience

East Asian and Caucasian children from North America and Europe show marked differences in EF development and are subject to different cultural and socialization influences. This has led to the idea that cross-cultural differences in EF might be attributable to differences in children’s socio-cultural background (Sabbagh et al., 2006; Oh and Lewis, 2008). Evidence that East Asian preschoolers, for example, reach developmental EF milestones as much as 6 months earlier than Caucasian preschoolers (Sabbagh et al., 2006), and are socialized from an early age to observe collectivist values, has led some to attribute their precocious EF development to the effect of practicing self-regulation during daily routines (Sabbagh et al., 2006; Oh and Lewis, 2008; Lan et al., 2011). This seemingly self-evident proposition is not altogether straightforward, however, as it assumes that EF development can vary as a function of social experience generally and practice specifically. This, it turns out, is a matter of considerable debate. On the one hand, interventions that target improving children’s EF and self-regulatory abilities have been designed to enhance their academic achievement (Diamond et al., 2007; Blair and Diamond, 2008; see Mattera et al., 2021 for review), implying that differences in children’s EF might originate, at least in part, from differences in children’s experiences (e.g., training). On the other hand, rigorously designed experimental efforts to improve EF through massive amounts of repeated practice yield no generalizable changes in underlying EF components (Redick et al., 2013; Simons et al., 2016; Kassai et al., 2019; Shepard et al., 2022). These latter findings are important as they directly challenge prevailing practice-based interpretations of the origins of cross-cultural differences in children’s EF (Sabbagh et al., 2006; Oh and Lewis, 2008).

Some of the tension in this debate appears to hinge on the conceptualization of EF itself (for recent discussions, see Doebel, 2020 and Zelazo and Carlson, 2022). Historically, EF has been defined as a set of domain-general cognitive processes that include working memory (Baddeley, 1992), inhibitory control (Diamond, 2013), and cognitive flexibility or shifting (Garon et al., 2008). To the extent that these processes were thought to operate the same way across different contexts (i.e., domain-general) but combine to support performance of individual tasks, EF came to be viewed as a fixed set of underlying dimensions or “components” of higher-order cognition (Miyake et al., 2000). However, it is precisely when EF is conceptualized and operationalized in this way that it proves to be largely resistant to the effects of practice.

A more recent conceptualization of EF, termed the control skills model, offers a potentially fruitful way forward (for discussion, see Doebel, 2020). On a control skills account, EF consists of a *set of skills for achieving goals*, skills that depend critically on knowledge, beliefs, values, norms, and preferences for *how* to use control to achieve goals in particular contexts. This account contrasts with the componential account that views EF as a domain-general process that functions in a stereotyped way across contexts. Imagine, for example, a child who needs to prepare for an upcoming math test but who wants to go out and play with their friends. From the traditional componential definition of EF, achieving control in this situation would involve a general capacity for inhibition, which would allow the child to halt their desire to play and remain focused on their work. From a control skills perspective, on the other hand, achieving control in this situation would involve an appeal to some form of knowledge which would help the child achieve their goal. The child might consider a social norm (e.g., most of my classmates will be studying so I should too), a value (e.g., doing well on the math test is important to me, so I should study), or knowledge about the punishment that would follow from a poor test result (e.g., my parents will withhold my allowance if I do not do well on the math test). Importantly, achieving control in this instance will depend critically on knowledge, values, and norms that are specific to this particular context and that have been acquired through a lifetime of socialization experiences. As it delineates pathways through which culturally unique social experiences could impact children’s EF, a control skills model of EF potentially offers important advantages over traditional componential models of EF for understanding the social origins of EF differences in early development.

However, even if we reconceptualize EF so that pathways linking social experiences and children’s EF can more plausibly be delineated, there are additional conceptual challenges associated with understanding cross-cultural differences in EF development. This has to do with the fact that associations between social influences, such as parenting characteristics, and child outcomes, such as self-regulation and the internalization of social rules, can vary across cultural contexts. Consider, for example, the link between authoritarian parenting styles and child self-regulation. Authoritarian parents demand compliance from their children and endorse the use of power assertion for achieving parenting goals but lack emotional warmth when interacting with their children (Baumrind, 1966, 1967). In Western cultures, authoritarian parenting is associated with lower social adjustment and self-regulation among children, at least as compared to children of authoritative parents (Dornbusch et al., 1987; Milevsky et al., 2007; Murray, 2012). In East Asian cultures, by contrast, authoritarian parenting is not consistently associated with negative child outcomes, but in selected studies, has been associated with child achievement and academic adjustment (Dornbusch et al., 1987; Steinberg et al., 1992; Leung et al., 1998; Chao, 2001; Ang and Goh, 2006; Kim and Park, 2006; Liu and Guo, 2010; Watabe and Hibbard, 2014; Pinquart and Kauser, 2018). Given that the same parenting behaviors can have different implications for children outcomes in different cultural contexts, understanding associations between social influences and children’s EF development will require careful consideration of the cultural meaning of parenting behaviors in different cultural contexts (for full discussion, see Chao, 1994). The critical point here is that the same parenting behaviors can have very different implications for the development of children’s EF when those behaviors are expressed in different cultural contexts. Thus, even if we allow for the possibility that social experience can impact EF development, we need to acknowledge that cultural context can moderate associations between social experiences and child outcomes.

## 3. A theoretical model relating culture, academic achievement, and executive functions

We return now to the question that opened the manuscript, namely whether the East Asian advantage in academic achievement compared to North American peers can be explained, at least in part, by differences in EF? At first blush, the connection seems almost self-evident. After all, early differences in EF predict academic achievement (Best et al., 2011; Jacob and Parkinson, 2015; Samuels et al., 2016; Ahmed et al., 2019; Robson et al., 2020), and children of East Asian origin excel on measures of EF (Sabbagh et al., 2006; Oh and Lewis, 2008; Lan et al., 2011; Imada et al., 2013; Wang et al., 2016; Ellefson et al., 2017; Cho et al., 2021). However, a closer examination of the hypothesis (i.e., cultural differences in EF → differences in academic achievement) reveals a variety of new questions and conceptual challenges. Why, for example, might children of East Asian origin show an advantage in EF? Although the common assumption is that the advantage takes root in East Asian children’s unique cultural and social environment, conceptually it is not clear how variation in social experience begets variation in EF. Moreover, links between socialization practices (i.e., parenting characteristics) and child outcomes can vary across cultural contexts.

**Figure 1:**
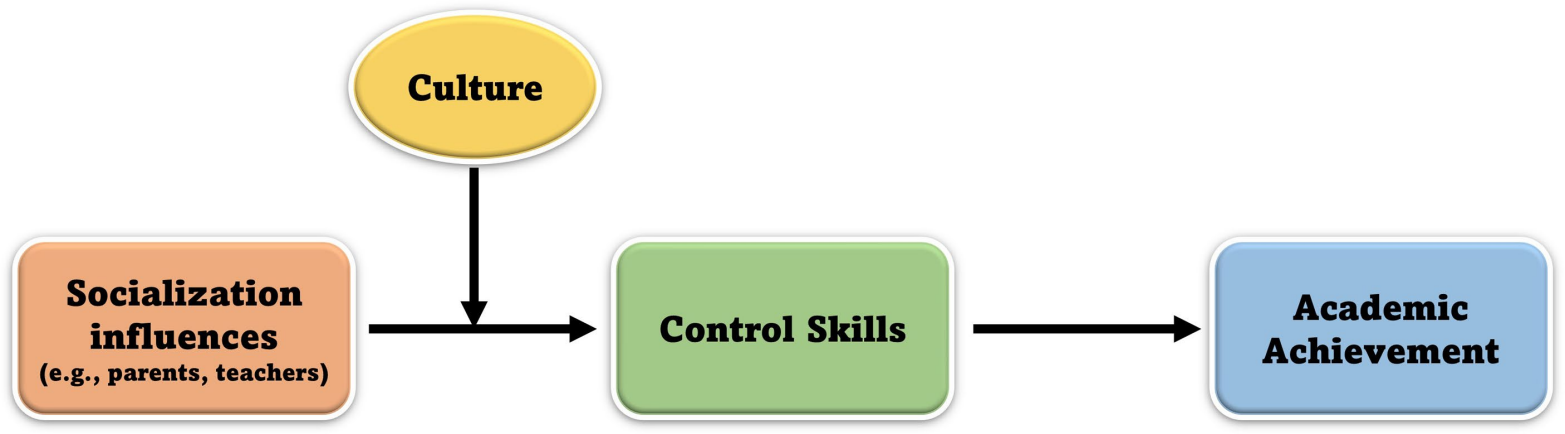
A diagram of the model on culture, academic achievement, and executive functions.

Despite these challenges, we believe that executive functions can, and indeed, should be integrated into models of cultural differences in academic achievement. Doing so will help synthesize findings from the fields of developmental, cross-cultural, and educational psychology, delineate potential pathways linking social experience to EF development and academic achievement, and potentially lead to new testable hypotheses. Here, we propose such an integrative model. A diagram of the model is shown in Figure 1. At the core of the model is the idea that children’s EF be conceptualized as a set of *control skills* for achieving goals (Doebel, 2020). This represents a departure from the way EF has historically been conceptualized in educational and cross-cultural studies, where the focus has been on underlying EF components such as working memory, inhibitory control, and mental flexibility. The model also draws a distinction between cultural and “social influences” on control skills. “Social influences” refer to the characteristics and behaviors of a child’s key socializing agents, including parents, grandparents, and teachers, whereas culture denotes the broader system of norms and values within which caregiver-child relationships and interactions are embedded. Relatedly, another important feature of the model is that “social influences” are portrayed as a proximal influence on children’s control skills whereas culture moderates the association between socialization factors and children’s control skills. Finally, the model identifies “control skills” as an important predictor of “academic achievement” as the existing evidence on EF and academic achievement suggests that EF predicts academic achievement (Latzman et al., 2010; Samuels et al., 2016; Ahmed et al., 2019; Robson et al., 2020). The model provides a framework for integrating findings from the fields of developmental, cross-cultural, and educational psychology, and helps to resolve important conceptual challenges related to such an integration with the following two main points.

First, the model lays out a conceptually plausible means by which social experiences influence children’s EF, a key challenge in characterizing social determinants of children’s cognitive development. It does so by adopting a *control skills* characterization of EF in lieu of a more traditional componential model of EF. As discussed, the key shortcoming for the componential view of EF is that there is little empirical evidence for far-transfer effects of training core EF components (e.g., inhibitory control, working memory, and shifting; Redick et al., 2013; Kassai et al., 2019; Shepard et al., 2022), and this contradicts with prevailing practice-based interpretations of the origins of cross-cultural differences in children’s EF. Control skills, by contrast, take root in and are shaped and influenced by the child’s social environment. With this conceptual groundwork in place, the model can accommodate findings concerning the socio-cognitive determinants of the East Asian advantage in academic achievement. One influential study on academic achievement, for example, combined data from two nationally representative longitudinal surveys to test whether East Asian student’s academic advantage relative to Caucasian students is attributable to differences in socio-economic status, intellectual abilities, or work ethic (Hsin and Xie, 2014). By virtue of the longitudinal nature of the data, the researchers were able to show that the emergence of the Asian-Caucasian achievement gap coincided with the divergence in Asian and Caucasian students’ beliefs about the importance of work ethic for academic achievement. Whereas Asian students were more likely to attribute academic success to work ethic, Caucasian students were more likely to attribute success to inborn ability. Finally, Asian students reported greater parental valuation of academic achievement compared to Caucasian students. Although the study was not explicitly framed in terms of EF, the findings are readily accommodated by the proposed control skills model in that differences in socialized beliefs and values contribute to differences in the deployment of control skills in academic settings (e.g., cultural practices of training, parental socialization of self-regulation, and parental valuation of academic effort). This in turn contributes to differences in academic effort and achievement (for related discussion, see Niebaum and Munakata, 2022).

Second, the model draws a distinction between social and cultural influences on EF with different roles. Social influences refer to the characteristics and behaviors of caregivers (e.g., parents and teachers), and they can impact children’s control skills directly. Cultural influences, by contrast, refer to the broader system of norms and values within which caregiver-child relationships are embedded and *moderate* the association between social influences and children’s EF. As such, the model can accommodate evidence that the same class of parenting attitudes and behaviors (i.e., authoritarian parenting) can have different associations with child outcomes in different cultural backgrounds (Dornbusch et al., 1987; Leung et al., 1998; Chao, 2001; Kim and Park, 2006; Murray, 2012; Pinquart and Kauser, 2018). In North America, restrictive and controlling parenting behaviors are often rooted in negative views of children, such as notions of original sin, and motivated by an interest in breaking the will of the child (Smuts and Hagen, 1985). In East Asia, by contrast, restrictive and controlling parenting behaviors can be viewed positively, as they are thought to reflect a commitment to family socialization (e.g., “*ga-jung-kyo-yuk*” in Korean; see Choi et al., 2013), training (e.g., “*chiao shun*” in Chinese), and governance (e.g., “*guan*” in Chinese). This form of parenting is considered important in East Asian cultures so that children can learn their position in society and help to maintain social harmony. Thus, parenting practices with firm control, training, and governance can appear controlling from a Western point of view, but in an East Asian cultural context, they can signal care, love, and concern for a child’s education, which is somewhat different from a solely “authoritarian” parenting styles (Leung et al., 2010; Huang and Gove, 2015). Whether culture moderates the association between parenting behaviors and children’s EF as predicted by the model has not been investigated directly but is certainly conceivable. Indeed, some have speculated that collectivist cultures make it easier for Asian-American parents to inculcate values related to academic achievement and a strong work ethic, precisely because in collectivist cultures, there is a shared expectation that children will conform to family expectations (for discussion, see Hsin and Xie, 2014). To summarize, these examples illustrate the idea that culture is as a set of beliefs and values that are jointly held by caregivers and children so that it should be understood in the corresponding cultural settings (Chao, 1994; Ang and Goh, 2006; Huang and Gove, 2015; Cheung and Lim, 2022). By having two separate entities (i.e., social influences as a variable influencing children’s EF and culture as a moderating variable between social influences and children’s EF), the model has merit in that it cannot only accommodate the findings on possible difference in psychological interpretation of certain socialization practices depending on cultures, but also allow our better understanding of *how* they impact children’s EF.

## 4. Implications for future research

The proposed model is an integration of ideas about the nature of EF, cultural differences in socialization experiences (e.g., parenting), and academic achievement. It is, of course, possible to imagine more basic models in which caregiver socialization impacts children’s academic achievement directly without any intervening influence of EF. Parents may encourage their children to expend greater effort on their studies, for example, which may have a direct influence on children’s achievement. Although greatly simplified, we feel that such models do not provide sufficient conceptual tools to delineate *how* children achieve success academically. Parental valuation of their children’s hard-work and attention to academic matters is undoubtedly important for children’s achievement in academic settings, but only to the extent these parenting strategies shape children’s goals (e.g., “My goal is to do well at school”) and engage their children’s motivation to achieve these goals (e.g., “I am prepared to work very hard to do well at school”). As such, it is important to include child characteristics of some kind in models relating parenting behaviors and children’s academic achievement. We believe control skills is a useful construct in this regard. The ultimate value of the model though is that it helps to identify research questions and generate predictions for future studies in this area.

First and foremost, the model raises questions about the measurement of EF. How, for example, should EF be measured in cross-cultural studies involving children? Historically, EF has been measured through the administration of age-appropriate working memory, flexibility, or inhibitory control tasks. This makes sense from a componential standpoint, as these tasks have been designed to yield relatively pure measures of underlying EF components or processes. From a control skills perspective, however, control is not founded on underlying domain-general components and the assumption that laboratory tasks generate pure measures of EF is considered suspect. From this standpoint, EF should be measured with procedures with greater ecological validity than standard “laboratory measures,” procedures such as non-computerized tasks that use real objects and scenarios that are reminiscent of the school context (for fuller discussion, see Doebel, 2020). It may also be promising to examine parent–child interactions and/or teacher-child interactions in the context of these procedures to shed light on how caregivers support children’s developing control skills. To date, however, ecologically valid procedures for examining control skills in academic contexts have not been developed or validated. The development of such measures therefore constitutes an important avenue for future work.

A related issue concerns the measurement of culture. Despite the number of studies that have examined cross-cultural differences in EF and academic achievement, there is little consensus about how culture should be measured. In fact, many such studies do not measure culture at all: participants are simply grouped according to their self-reported ethnicity (i.e., East Asian, Caucasian) or national origin (e.g., China, United States) on the assumption that any individual from a particular region or country possesses a particular set of cultural values (e.g., collectivist and individualist). This is problematic in a number of ways, not least of which that attributing effects to unmeasured independent variables seems decidedly risky from a scientific standpoint. A larger issue is that culture in regions like East Asia and North America is regionally quite varied and, in some cases, undergoing rapid change. To assume that all individuals within a region share the same cultural orientation is at odds with the complexity of cultural life in these regions. Indeed, studies that have measured culture directly do not find that individuals from East Asian countries are equally collectivistic, nor necessarily less individualistic than individuals from North America. For example, when comparing individuals from two putatively collectivist countries (i.e., Japan and South Korea), individuals from Japan typically view themselves as an indivisible part of larger social contexts (i.e., highly collectivistic), whereas individuals from South Korea typically view themselves as autonomous agents capable of influencing social contexts (i.e., relationalism; bi-directional dynamics between the self and contexts; see Park and Han, 2018 for discussion). A second study that compared EF in children from Canada and South Korea found that parents of South Korean preschoolers reported being more individualist and less collectivist in their values compared to parents of Canadian preschoolers (Cho et al., 2021). Findings from these studies are at odds with common assumptions about cultural orientations in East Asia and North America and underscore the importance of measurement of culture in cross-cultural research on EF.

Beyond issues of measurement, the proposed model raises questions about the relationship between parenting, academic achievement, and culture. According to the model, parenting style relates differently with child outcomes in East Asian and Caucasian samples because of the moderating influence of culture. But what aspect of culture exerts this moderating influence? The most common assumption is that East Asian and Caucasian cultures are best distinguished in terms of collectivist (or Confucian) vs. individualist values, respectively. While these value systems differ markedly with respect to the relative importance of the individual vs. the group, they are extremely general and not specific to the caregiver-child relationship or child education. Indeed, country-level individualism–collectivism does not always correspond to individual-level individualism–collectivism (see Li et al., 2018). Although at the group-level, East Asian cultures can be collectivistic and Western cultures can be individualistic, caregiver-child relationships at the level of the individual may differ from the group-level norms. Furthermore, the “East Asian” advantage in academic achievement, at least in the North American context, is not specific to children of parents from East Asian countries known for their Confucian value orientation — it also extends to children of parents from South Asian countries whose cultures are not distinctly Confucian (for discussion, see Hsin and Xie, 2014). Moving forward, it may therefore be more profitable to examine values more specifically related to parenting, child behavior, and school achievement and that more directly shape how caregivers and children interpret various social interactions related to children’s intellectual development and academic performance.

Finally, the proposed model raises questions about cultural differences in EF early in development and their implications for later academic achievement. The proposed model moves away from a componential view of EF in favor of a control skills framework, in part because EF components are not amenable to change through practice in the same way as control skills. But then why do children from East Asian countries typically outperform same-aged Caucasian children from North America or Europe on laboratory (i.e., componential) measures of flexibility, inhibitory control, and working memory? One possibility is that cross-cultural differences in children’s EF reflect differences in socio-cultural dynamics of psychological testing procedures rather than differences in underlying EF components. In studies of young children, testing is always done in person with an experimenter. However, children are not randomly assigned to experimenters in cross-cultural studies — it is not feasible. Instead, experimenters from one country typically test all children in their country and experimenters from the comparison country test children in their country. Consequently, cross-cultural differences in EF might reflect differences in the meaning of psychological testing both for the children being tested as well as the experimenters who are conducting the testing, and/or the different dynamics between the children and experimenters across countries. Development of control skills measures (e.g., measures controlling for or at least minimizing the possibly different impact of experimenters on children across countries) may help to test these possibilities. Assuming that control skills measures are eventually developed, one final set of questions concerns whether componential measures or control skills measures would have a stronger predictive relationship with academic achievement. Interestingly, although cross-cultural differences in EF have been documented empirically, it is not clear whether these differences predict differences in academic achievement. The proposed model predicts that control skills measures (i.e., ecologically valid control skills measures in academic contexts) would be more strongly associated with academic achievement than componential measures of EF, and that cross-cultural differences in EF would predict differences in academic achievement. Indeed, evidence that differences in effort are more predictive of academic achievement than differences in intellectual proficiency (Hsin and Xie, 2014) is consistent with this prediction. Direct tests of these predictions however await future testing.

## 5. Caveats and limitations

There are of course a number of caveats and limitations of the proposed model that need to be discussed. First, there are obviously many other factors influencing the development of children’s EF that are not represented in the model, such as genetic, demographic, neurophysiological, and lifestyle (e.g., diet, sleep, exercise, etc.) factors to name but a few. Moreover, it is conceivable that “social influences,” the one factor referenced in the model, is at least partially confounded with these unreferenced variables (e.g., Schmitt et al., 2019). Comprehensively assessing biological, lifestyle, and social influences is clearly important if we are to understand the unique impact of factors such as parenting attitudes and behaviors on EF development. At the same time, measuring and modeling such a broad range of measures in an adequately sized international sample is beyond the scope of most research groups, but it would require the coordinated efforts of a large international consortium [for examples, see the Adolescent Brain Cognitive Development^SM^ (ABCD) Study, the Generation R study, and Growing Up in Singapore Toward healthy Outcome (GUSTO) birth cohort study].

A second limitation of the model is that it does not explicitly consider the difference between East Asian children growing up in East Asian countries and children of East Asian immigrants growing up in Europe or North America. Most cross-cultural studies of EF development compare Caucasian children residing in North America/Europe and East Asian children residing in East Asian countries (Sabbagh et al., 2006; Oh and Lewis, 2008; Lan et al., 2011; Ellefson et al., 2017; but see Yang et al., 2011 and Cho et al., 2021 for exception including East Asian immigrant children in North America). The strongest empirical evidence for the “East Asian” advantage in academic achievement, however, comes from national longitudinal studies that compare East Asian immigrant and Caucasian non-immigrant students on a common set of academic outcome measures (Hsin and Xie, 2014; Feliciano and Lanuza, 2017). International comparisons of academic achievement can be more challenging as there are fewer common outcome measures available and a greater potential for unmeasured sources of variability between samples. That said, comparisons of academic achievement of East Asian immigrants and Caucasians (North American or European) confound immigrant status with socio-cultural factors specific to the East Asian community. This is an important limitation of the current model given that the immigrant experience itself profoundly impacts the social dynamics of the family and children’s achievement in academic settings (Fuligni, 1997). Whether socio-cultural influences unique to East Asia can be distinguished from the effects of immigration of course remains unclear, and it is possible that the current model could be adapted to accommodate both influences. Given the current state of the literature, however, it was not possible to explicitly distinguish between the effects of culture and immigration on children’s academic achievement.

## 6. Summary and conclusion

In the proposed model, culture acts as a moderating variable that modifies caregiver socialization on children’s cognition, rather than a fixed variable that directly influences child cognition without considering the possibility of different context-specific social influences on child cognition. Although it is possible that culture itself can be an antecedent variable that influences both socializing agents and children’s control skills, the proposed model can accommodate evidence that the same class of parenting attitudes are associated with different child outcomes—presumably because the same class of parenting attitudes are interpreted differently by children in different cultural contexts. The proposed model is a first-step in elucidating *how* socializing agents, culture, EF control skills, and academic achievement are related to each other with a more nuanced explanation, rather than simply assuming that culture impacts EF and ultimately influences academic achievement. A consideration of cultural factors in understanding links between EF and education is imperative for revealing whether observed effects are universal or culture specific. These considerations are particularly timely given that the majority of existing research in the field of psychology is based on WEIRD (Western, Educated, Industrialized, Rich, and Democratic) participants (see Gutchess and Rajaram, 2022 for theoretical review). Therefore, the proposed model may provide further insights into the underlying the relationship between socializing agents, culture, EF control skills, and academic achievement in diverse contexts.

## Data availability statement

The original contributions presented in the study are included in the article/supplementary material, further inquiries can be directed to the corresponding author.

## Author contributions

JBM, IC, and NH-K contributed to conception and wrote the first draft of the manuscript. All authors contributed to the article and approved the submitted version.

## Funding

This work was supported by funding from the Social Sciences and Humanities Research Council of Canada (grant number: R3353A12; awarded to JBM).

## Conflict of interest

The authors declare that the research was conducted in the absence of any commercial or financial relationships that could be construed as a potential conflict of interest.

## Publisher’s note

All claims expressed in this article are solely those of the authors and do not necessarily represent those of their affiliated organizations, or those of the publisher, the editors and the reviewers. Any product that may be evaluated in this article, or claim that may be made by its manufacturer, is not guaranteed or endorsed by the publisher.
